# Accumulation of Amyloid Beta (Aβ) Peptide on Blood Vessel Walls in the Damaged Brain after Transient Middle Cerebral Artery Occlusion

**DOI:** 10.3390/biom9080350

**Published:** 2019-08-08

**Authors:** Antonio Henrique Martins, Astrid Zayas-Santiago, Yancy Ferrer-Acosta, Solianne M. Martinez-Jimenez, Lidia Zueva, Amanda Diaz-Garcia, Mikhail Inyushin

**Affiliations:** 1 Pharmacology and Toxicology Department, University of Puerto Rico, Medical Sciences Campus, Guillermo Arbona, Área de Centro Médico Río Piedras, PR 00935, USA; 2 Department of Physiology, Universidad Central del Caribe Ave. Laurel #100, Santa Juanita, Bayamón, PR 00956, USA; 3 Department of Neuroscience, Universidad Central del Caribe Ave. Laurel #U26, Santa Juanita, Bayamón, PR 00956, USA

**Keywords:** ischemic stroke, middle cerebral artery occlusion, amyloid, Aβ peptides, blood vessels

## Abstract

It is well known that amyloid beta (Aβ) peptides are generated in blood vessels, released into the brain during thrombosis, and temporarily accumulate in this organ after injury. Here we demonstrate that 24 h after transient middle cerebral artery occlusion (tMCAO), one of the standard models of focal ischemic stroke, Aβ peptide accumulates in the brain, concentrating on the blood vessel walls. Because Aβ oligomers are known to induce significant damage to brain cells, they act as an additional damaging factor during ischemic stroke. Considering that they have been shown to form ion channels in cells, affecting osmotic balance, we used an Aβ peptide channel blocker, tromethamine (2-amino-2-(hydroxymethyl) propane-1,3-diol), to prevent this additional injury. Tromethamine injected 0.1 g/100 g body weight intraperitoneally at 5 min before tMCAO decreased water content in the damaged hemisphere, as measured by dry brain weight. Congo red staining, which binds only to Aβ oligomer plaques (amyloid), showed that there was no significant presence of plaques. Therefore, we suggest that Aβ peptide oligomers are responsible for some of the brain damage during stroke and that blockage of the ion channels that they form could be beneficial in treating this complex neurological syndrome.

## 1. Introduction

Amyloid beta (Aβ) peptides became well known after being established as the main component of polymeric amyloid plaques in Alzheimer’s disease (AD) [1]. Later, the accumulation of Aβ in tissue was found to be a common factor in several health problems, including cancers, glaucoma, brain trauma, pre-eclampsia, and often during thrombosis (reviewed in [2,3]). Transient Aβ plaques were observed in the brain of an AD mouse model after mild brain trauma, and these plaques became undetectable after seven days. During this post-traumatic period, the presence of Aβ plaques was also correlated with the presence of soluble Aβ oligomers in the brain [4]. Multiple studies have confirmed that Aβ plaques and oligomers are detected in the brain of human patients within hours of traumatic brain injury (TBI) in non-AD patients [5,6,7]. We have shown recently that coagulation produces a massive local release of Aβ from thrombotic blood vessels during experimental photothrombosis in the brain or in the skin [8,9]. Thrombocytopenia significantly reduces Aβ production during thrombosis, again implicating platelets as major players in this process [8]. Previously, it was shown that platelets are the main source of Aβ in the blood [10], and platelet α-granules contain amyloid precursor protein (APP), which is released during degranulation [11,12,13]. To produce Aβ peptides, soluble extracellular APP from platelets may be partially processed at a later time point (reviewed in [2]). The pathway for Aβ processing from released soluble APP is different from the neuronal-type, membrane-bound process and involves a regulated secretory vesicle pathway in which the specialized cathepsin B enzyme works as a β-secretase [14,15,16]. It has been suggested that a major portion of secreted extracellular Aβ peptides is produced in this pathway [17]. 

It is important to note that one of the Aβ peptide’s proposed functions is to act as an antimicrobial agent in the innate immune system [8,18,19] (reviewed also in [2,3,20,21]). Aβ peptide has a strong antimicrobial activity against bacteria, fungi, and viruses [18,22,23,24], and mice with AD show increased protection against bacterial infection [19]. We have shown that, during thrombosis in skin blood vessels, Aβ peptides are generated and released in the nearby skin tissue. This peptide also works as a pore-forming antibiotic against fungal cells [9]. While there may be a variety of other antibiotic mechanisms, it was shown previously that soluble Aβ peptide oligomers at low concentrations perforate cell membranes by making tetrameric channels penetrable by K^+^ ions, and at higher concentrations these peptides form Ca^2+^-permeable hexameric pores [25,26,27]. An excess of Ca^2+^ flux through these pores induces extremely toxic calcium dyshomeostasis [28,29,30]. This channel pore-forming mechanism is already known in peptide antibiotics such as nystatin and amphotericin and many natural peptide antibiotics [31,32]. We suggest that this Aβ antibiotic may be generated by platelets as a response to tissue damage, inflammation, and infection [2,3], as these are natural causes of platelet activation and degranulation [9]. In many pathological conditions, however, additional Aβ may aggravate the situation. 

Thus, our study provides novel insight into Aβ peptide generation and its relation to vessel damage and inflammation following ischemic stroke. While it has been reported that circulating Aβ peptide levels are elevated in patients with acute ischemic stroke and vascular lesions [33,34], this condition has never before been attributed to the release of Aβ by platelets during decreased circulation in the brain. In this report, we used transient middle cerebral artery occlusion (tMCAO) to test whether Aβ peptides accumulate in the brain after this neuroinflammatory insult, and we investigated the possible therapeutic strategy of blocking the Ca^2+^-permeable pores formed by Aβ with the specific blocker tromethamine (Tris buffer) [35] to decrease the subsequent damage.

## 2. Materials and Methods

### 2.1. Ethics Statement

All procedures involving rodents were conducted in accordance with the National Institutes of Health (NIH) regulations concerning the use and care of experimental animals and approved by the Universidad Central del Caribe Institutional Animal Care and Use Committee (IACUC). Surgical procedures were performed using sterile/aseptic techniques in accordance with institutional and NIH guidelines. To minimize discomfort, animals were anesthetized in all procedures involving surgery and before euthanasia.

### 2.2. Animals

Male and female Sprague Dawley rats weighing 250–300 g (3 to 4 months old) from the Universidad Central del Caribe Animal Facility were used in our experiments. All animals were subjected to brain ischemia. The experimental group was injected intraperitoneally with tromethamine (Tris-HCl, 0.1 g/100 g body weight, pH 7.4, in 0.9% sterile saline), whereas the control group was injected with an equal volume of 0.9% sterile saline solution. 

### 2.3. Transient Middle Cerebral Artery Occlusion (tMCAO) 

Animals were subjected to left-hemisphere transient middle cerebral artery occlusion (tMCAO) using the intraluminal suture technique, as previously described by Martins et al. [36]. Briefly, anesthesia was induced with 5% isoflurane, and the animals were kept anesthetized using a mixture of gases (2% isoflurane, 700 cc/min NO_2_, and 300 cc/min O_2_) using a vaporizer for small animals (Rockmart, GA, USA) in a breathing mask during the entire surgical procedure. Rat core temperature was continuously monitored and maintained in the range 36.5–37.5 °C using a DC Temperature Control System (FHC, Bowdoin, ME, USA) with a built-in rectal thermometer and heating pad.

To monitor cerebral blood flow in the middle cerebral artery (MCA) throughout the procedure, a laser Doppler instrument (PeriFlux 5000, Perimed, Stockholm, Sweden) was placed over the skull at 1 mm posterior and 3 mm lateral to bregma. To induce ischemia, the common carotid artery was exposed through an incision in the neck (above the front paws). The superior thyroid and occipital artery were cauterized using a small-vessel cauterizer (Fine Science Tools, Foster City, CA, USA), and the pterygopalatine artery was tied with a 6-0 nylon monofilament suture thread (Fine Science Tools) to avoid blood reflux and allow easier entry of the 4-0 silicone tip nylon filament (Doccol Corp., Sharon, MA, USA) into the interior carotid artery. Micro-serrefine (clamp) vessel clips (Fine Science Tools) were placed at the base of the common carotid artery and the internal carotid artery to momentarily stop blood flow. A small incision was made to the external carotid artery to insert the 4-0 silicone tip nylon filament into the internal carotid artery up and toward the MCA until a drop in blood flow was observed. A cerebral blood flow reduction of 65% or more from the initial normal blood flow was considered a successful stroke. Transient ischemia was induced for 1 h with the insertion of the filament. After this period of time, the filament was removed, and as a consequence the blood flow was restarted (reperfusion), as visualized using a laser Doppler instrument. During the recovery period, animals were kept at 32 °C. To ensure comparable results, only animals with successful stroke and similar drops (± 5%) in cerebral blood flow were compared.

### 2.4. Rat Brain Preparations for Immunofluorescence after tMCAO 

Twenty-four hours after tMCAO surgery, the animals were heavily anesthetized with isoflurane and transcardially perfused using a perfusion fixative solution of 4% paraformaldehyde and 4% sucrose dissolved in 0.1 M phosphate-buffered saline (PBS: NaCl, 137 mM; KCl, 2.70 mM; Na_2_HPO_4_, 10.14 mM; KH_2_PO_4_, 1.77 mM; pH 7.2). Following perfusion fixation, the brains were extracted and incubated overnight (16–24 h) in the same fixative solution (40 mL), with mild agitation. After incubation the tissue was transferred to PBS. Samples were cut (40 μm thickness), and some slices were stained for “ischemia contrast” by NeuroScience Associates (NSA, https://www.neuroscienceassociates.com) to mark the damaged zone. Adjacent brain slices without staining by NSA were used to perform immunofluorescence with antibodies against amyloid protein (Aβ) and for electron microscopy. 

### 2.5. Immunofluorescence and Confocal Microscopy

Immunostaining was performed using a previously established protocol [37,38]. First, the 40 µm sections were treated for 20 min with a permeabilization solution consisting of 0.03% Triton X-100 (Sigma-Aldrich, St. Louis, MO, USA) and 1% dimethyl sulfoxide (DMSO; MP Biomedicals, Santa Ana, CA, USA) in 0.1 M PBS under gentle agitation (40 rpm). Second, the sections were treated for 60 min with a blocking solution containing 5% normal goat serum, 5% normal horse serum (Vector Laboratories, Burlingame, CA, USA), and 2% *w/v* bovine serum albumin (BSA; Sigma-Aldrich) in permeabilization solution. Following the blocking step, the sections were processed separately using antibodies against Aβ. A primary rabbit polyclonal antibody against Aβ (1:400; Abcam, Cambridge, MA, USA, cat. #ab2539) was diluted in blocking solution and incubated for 16 h at 4 °C. Following three washes with permeabilization solution, the sections were incubated for 2 h at 25 °C with a goat anti-rabbit secondary antibody conjugated with Alexa Fluor 647 in blocking solution (1:400; Abcam, Cambridge, MA, USA, cat. #150083) while protected from light. The sections were then washed three times with 0.1 M PBS for 10 min and once with distilled water. 

Counterstaining with Congo red was performed as follows. The brain sections were transferred onto glass slides and allowed to air dry completely. The slides were first washed with 70% ethanol for 1 min, followed by 80% ethanol for 1 min, and then incubated for 15 min in a filtered solution of Congo red (0.22 µm filter). A 1% Congo red solution was prepared in 80% ethanol. After incubation, the slides were washed with 80% ethanol for 1 min followed by 70% ethanol for 1 min, washed twice with distilled water, and finally allowed to air dry completely. The glass slides were mounted with Fluoroshield mounting medium containing 4′,6-diamidino-2-phenylindole (DAPI; Sigma-Aldrich, cat. #F6057).

Images were acquired using an Olympus Fluoview FV1000 scanning inverted confocal microscope system equipped with a 4×, 10×, 20×, or 40×/1.43 oil objective (Olympus, Melville, NY, USA). The images were analyzed using ImageJ software (ver. 1.8.0_112 (http://imagej.nih.gov/ij) with the Open Microscopy Environment Bio-Formats library and plugin, allowing for the opening of Olympus files (http://www.openmicroscopy.org/site/support/bio-formats5.4/). The images were evaluated using custom colorization.

### 2.6. Electron Microscopy

A small part of the 40 μm brain sections containing the damaged area caused by ischemia (confirmed by staining of adjacent sections with NSA contrast marking) was fixed in 2.5% glutaraldehyde, 4% paraformaldehyde in 0.09 M cacodylate buffer with 0.2 mM CaCl_2_ for 1.5 h at 5 °C, washed with 0.09 M sodium cacodylate buffer, and postfixed in 1% osmium tetroxide (OsO_4_) with 1.5% KFeCN in the same buffer for 30 min. After treatment with 1% OsO_4_ for 30 min, the ultrathin slices were then incubated in a 2% aqueous solution of uranyl acetate (UO_2_(CH_3_OCO)2·2H_2_O) for 1 h and washed. After dehydration through a graded series of acetone–water mixtures, the slices were embedded in Epon/Spurr epoxy resin. Ultrathin sections of 50–60 nm were obtained using a Leica Ultratome (Leica Microsystems, Wetzlar, Germany) and examined with a JEM100CXII electron microscope (JEOL Ltd., Tokyo, Japan).

### 2.7. Measuring Water Content and Blood–Brain Barrier Permeability in Rats after tMCAO

To test the effect of Tris (tromethamine), a blocker of Ca^2+^-permeable pores formed by Aβ, on brain water content and blood–brain barrier (BBB) permeability, eight rats were used. Four control rats were treated with 0.9% saline, and four rats were treated with Tris-HCl. Five minutes before tMCAO, the rats were injected intraperitoneally with 0.1 g/100 g rat weight of Tris-HCl in 0.9% NaCl, pH 7.4. After surgery, the animals were injected intraperitoneally [39] with 2% Evans blue (4 mL/kg) to measure BBB permeability. Twenty-four hours after surgery, the animals were anesthetized and quickly decapitated. The brains were removed, divided into hemispheres using a blade, and placed on a plastic weighing boat to weigh and dry. Brains were left to dry at 50 °C until the weight of the tissue remained constant. The percentage of brain water content after the tMCAO was determined using the following expression: 100 × (wet weight − dry weight)/wet weight. 

To measure BBB permeability, the Evans blue content in the brains was determined. Dry brains were weighed on day 12, divided into six sections with a blade to allow better Evans blue solubilization, and placed inside centrifuge tubes. Formamide (500 μL) was added to each dry brain sample, which was then transferred to a 55 °C water bath and incubated for 48 h. Samples were centrifuged (1000× *g* for 5 min), and the supernatant was removed. The absorbance was measured at 610 nm using a spectrophotometer (Versa Max tunable micro plate reader, Molecular Devices, Downingtown, PA, USA).

### 2.8. Chemicals and Materials

All other chemicals and materials not specially mentioned in previous paragraphs of Materials and Methods were purchased from Sigma-Aldrich (St. Louis, MO, USA). 

### 2.9. Statistics and Measurements

GraphPad Prism 7.03 (GraphPad Software, Inc., La Jolla, CA, USA) was used for calculations of ordinary one-way ANOVA, Kruskal–Wallis, and post-hoc tests to determine statistical differences, as indicated for each experiment. Values were determined to be significantly different if the *p*-value was <0.05.

## 3. Results

### 3.1. After Focal Ischemic Stroke, Many Blood Vessels in the Damaged Zone Contained Coagulated Blood with Partially Degranulated Platelets 

**Figure 1:**
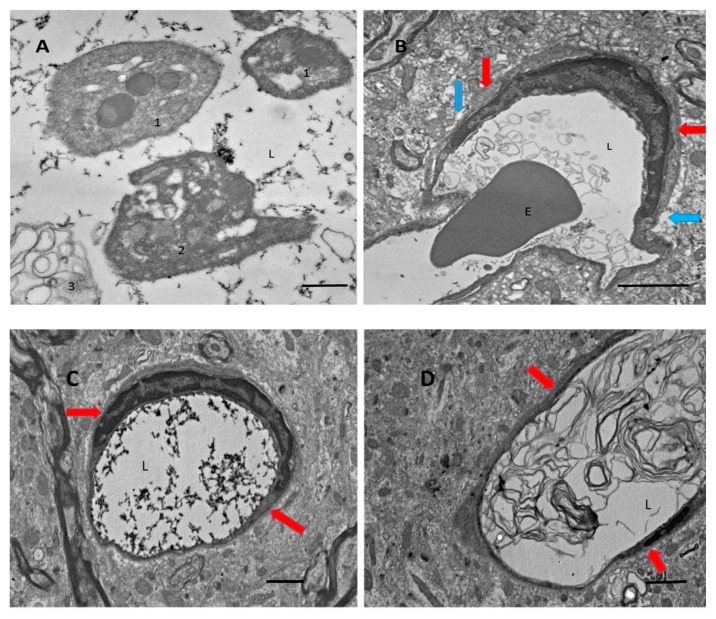
Blood vessels after transient middle cerebral artery occlusion (tMCAO) under the electron microscope. (**A**) Platelets, rounded and stellate (see text). (**B**) Erythrocyte (E) entrapped in debris. (**C**) Fibrin strands in the vessel lumen (L). (**D**) Significant swelling and vacuolar bubbles in the astrocyte endfeet around the blood vessels (blue arrows). Perivascular space (red arrows). Scale bars: (**A**), 500 nm; (**B**), 2 µm; (**C**,**D**), 600 nm.

Previously, we found that there is a massive release of Aβ during thrombosis [8]. In this study, we used electron microscopy to analyze blood vessels within the affected areas of the brain after ischemic stroke, and we looked for characteristic signs of coagulation. We found that vessels, especially small vessels, are filled with clot-related elements, such as fibrin strands (Figure 1A,C), clot-related debris (Figure 1B,D), and different forms of platelets (Figure 1A). Platelets also showed clot-related morphological conversions, from a relatively rounded form (Figure 1A, “1”), to stellate (Figure 1A, “2”), and then to a fully degranulated form (Figure 1A, “3”). Fibrin strands fill the majority (≈60%) of blood vessels in the ischemic zone (Figure 1C, also at high magnification in Figure 1A). Many vessels are also filled with debris that looks like lipid membrane bubbles (Figure 1B,D). Astrocyte endfeet, which envelop blood vessels walls in many cases, appear swollen (Figure 1, blue arrows), whereas basal membranes in perivascular spaces are expanded in volume (Figure 1, red arrows).

After we confirmed by electron microscopy that coagulation is present in the area affected by ischemia, and based on our previous research showing massive generation of Aβ peptides during photocoagulation, we hypothesized that Aβ might also be generated in damaged blood vessels after monofilament-induced ischemia.

### 3.2. Aβ Accumulates in Blood Vessel Walls and in Nearby Brain Tissue after tMCAO 

**Figure 2:**
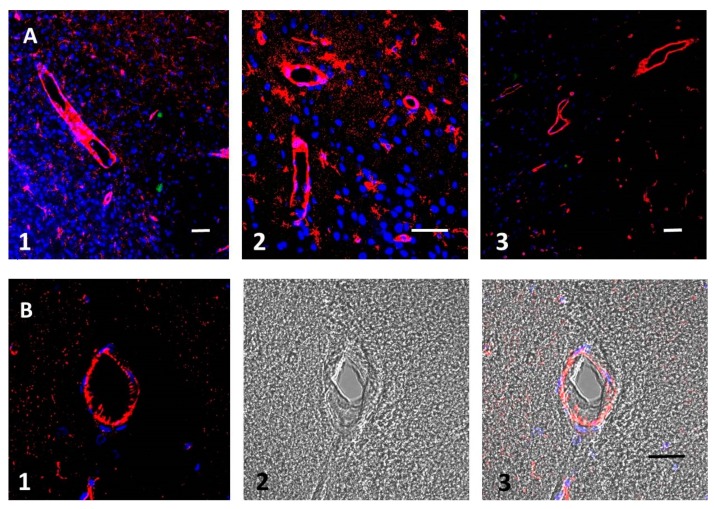
(**A**) Brain zone affected by ischemia. Amyloid beta (Aβ) peptide oligomer immunostaining (red), Congo red staining specific for aggregated amyloid (green). The walls of both large and small vessels are visible. Nuclear DAPI staining (blue) shows that Aβ does not mainly coincide with cells, whereas red staining is mainly present in blood vessels. Congo red staining specific for aggregated amyloid (green) is present as low-density spots in the tissue and not associated with any nucleus. (**B**) Cross-section of a blood vessel in the affected zone after stroke: (1) fluorescence, (2) bright field, (3) merged view. Scale bars, 100 µm**.**

To visualize the presence of Aβ peptides after transient ischemic stroke, we used highly specific anti-Aβ antibodies with low reactivity to aggregated forms or to amyloid precursor protein (APP). Immunostaining showed that these peptides are present in ischemic brain tissue (Figure 2A,B, red), whereas in sham-operated naïve (control) animals there was no Aβ immunostaining (Figure S1, Supplementary Materials). In these experiments, we also used additional tissue staining for aggregated amyloid, using amyloid-specific Congo red dye (green fluorescence). Nuclei were marked blue with 40,6-diamidino-2-phenylindole (DAPI).

Aβ immunofluorescence was concentrated in the blood vessel walls (Figure 2A,B) in both larger vessels and small capillaries, but in some areas Aβ can be observed inside brain tissue near the vessels and resembles the processes of small cells (Figure 2A, note scale bar). Aggregated amyloid (revealed by Congo red staining) was also visible in ischemic tissue as low-density green spots (Figure 2A, panel 1 and Figure 2A, panel 3). Nuclei (blue) are not coincident with Aβ or aggregated amyloid staining, suggesting that brain cell bodies are not involved in Aβ accumulation (see also confocal 3D movie of Figure 2A panels 1 and 2 and as Figure S2 and S3, Supplementary Materials). Distribution of Aβ in different animals had the same pattern (Figure 2A, panels 1–3), and in all cases the majority of Aβ is concentrated at the vessel walls, resembling the distribution of Aβ peptide in cerebral amyloid angiopathy (CAA), which is common in AD [40] (for a review see [2]). 

Because in CAA the Aβ deposits are known to be concentrated in the basal lamina of the perivascular space, we were looking for cross-sections of blood vessels to see whether Aβ was deposited similarly in our experiments. Figure 2B shows the cross-section of a blood vessel after stroke in the ischemia-affected zone, which shows that the distribution of Aβ immunofluorescence is concentrated mainly on the external surface of blood vessel walls.

In sham-operated (control) animals without tMCAO, there was no visible accumulation of Aβ in the brain (see Figure S1, Supplementary Materials). In our experiments, we confirmed that Aβ immunofluorescence is present after tMCAO in damaged zones and deposited mainly on the surface of blood vessel walls, suggesting that there is a massive release of Aβ after tMCAO.

### 3.3. Tromethamine Reduces the Effects of tMCAO on the Brain

It is known that Aβ can form nonselective ion channels in the external cellular membrane of brain cells, producing dyshomeostasis, synaptic dysfunction, and osmotic changes leading to edema and cell death. If Aβ generated during ischemic stroke can damage neurons and other brain cells, blocking these channels might help cells to survive and to reduce swelling. The polyamine tromethamine (also known as Tris, a popular buffer) is a reversible blocker of Aβ that forms Ca^2+^-permeable pores [35], and we therefore used it in our experiments. 

**Figure 3:**
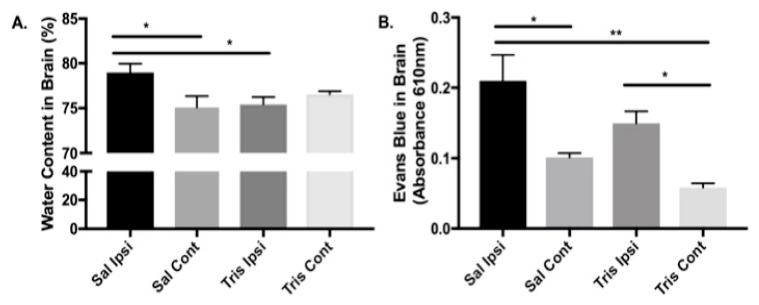
Percentage water content and Evans blue extravasation 24 h after tMCAO. (**A**) Water content of brain hemispheres in Tris- and saline-treated rats. Rats were treated with Tris-HCl (0.1 g/100 g rat weight, *n* = 4) or 0.9% NaCl saline (Sal, *n* = 4) and then subjected to tMCAO 5 min after treatment. The water content in each brain hemisphere 24 h after tMCAO was calculated for each treatment. Statistics: Kruskal–Wallis test and Dunnett’s multiple comparisons post-hoc test between hemispheres, Sal ipsilateral vs. Sal contralateral, * *p* = 0.027; Sal ipsilateral vs. Tris ipsilateral, * *p* = 0.035 Sal ipsilateral vs. Tris contralateral, *p* = n.s. (**B**) Evans blue infiltration in brains 24 h after ischemic stroke in Tris-HCl vs. saline-treated rats. Rats were treated with Tris-HCl (0.1 g/100 g rat weight, *n* = 4) or 0.9% NaCl saline (*n* = 4) and then subjected to tMCAO 5 min later. Evans blue infiltration content was obtained by measuring absorbance at 610 nm. Statistics: one-way ANOVA, Tukey’s multiple comparison post-hoc test between hemispheres: Sal ipsilateral vs. Sal contralateral, * *p* = 0.014; Sal ipsilateral vs. Tris contralateral, ** *p* = 0.001; Tris ipsilateral vs. Tris contralateral, * *p* = 0.038; Sal contralateral vs. Tris ipsilateral, n.s.; Sal ipsilateral vs. Tris ipsilateral, n.s.; Sal contralateral vs. Tris contralateral, n.s. Error bars represent the standard error of the mean; n.s., not significant.

Edema from excessive water content in the brain is a major contributor to poor outcomes following ischemic stroke [41]. Water content, expressed as g water/g dry weight as a percentage, is a standard method to measure these characteristics of brain damage. To verify whether tromethamine reduces water content inside the brain of animals with focal ischemia, the animals were treated 5 min before occlusion with either tromethamine (Tris)-HCl (0.1 g/100 g rat weight, *n* = 4) or an equal volume of 0.9% NaCl saline (*n* = 4) and then subjected to left-hemisphere tMCAO. Twenty-four hours after tMCAO, the water content in each brain hemisphere was calculated for each treatment. It was found that there was a significant reduction (*p* < 0.05) of water content in the ischemic ipsilateral hemisphere of tromethamine-treated animals compared with saline-treated controls (Figure 3A). This result suggests that tromethamine helps to maintain brain homeostasis by reducing water content in the ischemic hemisphere and thus reducing brain edema after ischemic stroke. Exact mean values with SEM error bars are as follows: the saline-treated ipsilateral hemisphere had 78.97% ± 0.97 water, whereas the Tris-treated ipsilateral hemisphere had 75.42% ± 0.82 water; the saline-treated contralateral hemisphere had 75.07% ± 1.24 water, whereas the Tris-treated contralateral hemisphere had 75.54% ± 0.36 water (*n* = 4, Kruskal–Wallis test, followed by Dunnett’s multiple comparisons test with the saline ipsilateral hemisphere, * *p* < 0.05).

To determine whether vasogenic edema after tMCAO was affected by tromethamine, we studied the infiltration of albumin from blood into brain using the Evans blue protein tracer method. It is known that Evans blue dye attaches mostly to blood albumin, and because albumin is a large molecule, it cannot cross a healthy BBB into the brain parenchyma. Thus, the presence of this dye in brain tissue suggests damage to BBB integrity, which permits entry of peripheral molecules into the brain after stroke. Brains from control and tMCAO-induced rats were dried and Evans blue extracted into the supernatant by formamide. The concentration of Evans blue in the supernatant was measured at 610 nm absorbance (Figure 3B). Our finding suggests that ischemic injury in the ipsilateral hemisphere causes damage to BBB permeability, which significantly (*p* < 0.05) affects Evans blue content inside the ischemic ipsilateral hemisphere compared with the contralateral hemisphere (Figure 3B). The difference in infiltration into the brain between the hemispheres decreased in tromethamine-treated animals (*p* = 0.0599), which may ultimately be biologically important, although it was not statistically significant in this experiment. The mean values with SEM error bars are as follows: saline-treated ipsilateral hemisphere, 0.2096 ± 0.0367; Tris-treated ipsilateral hemisphere, 0.1496 ± 0.01705; saline-treated contralateral hemisphere, 0.1015 ± 0.005977; Tris-treated contralateral hemisphere, 0.05815 ± 0.005977 (*n* = 4, one-way ANOVA, followed by Tukey’s multiple comparisons test, * *p* < 0.05, ** *p* < 0.01). These values suggest that the effect of tromethamine on cerebral edema after tMCAO is not directly mediated by the preservation of BBB integrity but clearly reduces the water-content effects of tMCAO in the damaged hemisphere.

## 4. Discussion

The present work established the presence of Aβ peptide in the ipsilateral part of the brain after tMCAO. Using electron microscopy, we also confirmed that in the damaged brain regions following tMCAO, the majority of small blood vessels showed signs of coagulated blood within and showed a clear swelling of astrocyte endfeet surrounding the blood vessels. This coagulation could be a source of Aβ, as was proposed elsewhere (see review in [2]). Blood coagulation produces massive amounts of Aβ peptides from the amyloid precursor protein (APP) liberated from platelets during experimental photothrombosis of brain blood vessels [8] and during experimental thrombosis of blood vessels in the skin [9] (Figure 1A–D). Aβ peptides are abundantly accumulated on the walls of small blood vessels, most likely on the external walls (Figure 2A,B), in some capillaries, and probably in astrocyte endfeet (Figure 2A). Our experiments showed that Aβ is present mainly in its non-aggregated form, which is recognized by a specific anti-Aβ-antibody, whereas only a small amount of aggregated Aβ, which is stained with Congo red, was detected in the form of isolated extracellular plaques (Figure 2A, panel 1, green spots). 

Previous reports have found that circulating Aβ peptide levels are elevated in patients with acute ischemic stroke [33,34]. Additionally, a time-dependent disappearance of diffuse amyloid plaques was discovered in ischemic brain [42], and this finding accords with those reported here. In general, brain damage after traumatic brain injury (TBI) is complicated by Aβ plaques and oligomer accumulation in the damaged part of the brain within hours of injury [5,6] (also reviewed in [7]). The cysteine protease cathepsin B has been identified as a powerful β-secretase, and its inhibition reduces Aβ production from its precursor APP [15,16,43]. It was shown that treatment with a small-molecule inhibitor of cathepsin B immediately after TBI resulted in recovery, with significant improvement in TBI-mediated motor dysfunction, reduced brain lesion volume, greater neuronal density in brain, and a reduction of apoptotic markers [17,43]. All these findings suggest that Aβ is an additional detrimental factor in brain damage from TBI and stroke. 

There are different ways in which a high rate of Aβ release could produce damage to the brain. (1) Higher concentrations of Aβ oligomers induce pore formation in the membranes of bacteria and fungi but at higher concentrations also in brain cells, producing cellular degeneration [25,44], and this damage may also extend to vessel cells [45]. (2) Aβ oligomers can produce vascular dysregulation by acting on pericytes [46]. (3) In addition, the presence of vascular amyloid alters water content, producing swelling in astrocytes and their endfeet [47]. Theoretically, the destructive effects could be diminished by reducing the production of Aβ oligomers during coagulation or by blocking the Aβ oligomer’s pore-forming effect. Thus, we asked whether the known blocker of Aβ-formed pores, tromethamine [35], could reduce the excess Aβ pore-forming effect and limit the additional pore-based damage during ischemia and coagulation following tMCAO. 

Tromethamine decreases water content in BBB leakage after tMCAO, as measured by analysis of the percentage of water in the brain, and does not significantly affect the extravasation of Evans blue (Figure 3A,B). This finding suggests that the preservation of BBB integrity is not involved in this effect of tromethamine. Previously, tromethamine was used as a treatment for ischemic stroke, but its effects were mainly attributed to its buffering “anti-edema” function [48]. This study proposes new connections between ischemic stroke and AD. Vascular problems and AD are clearly related, and both are age dependent (reviewed in [2,3]). Nearly half of the patients with AD have evidence of stroke in their brains [49]. In general, patients with AD have about a 200% higher risk of stroke, as was shown in a large cohort of AD patients in Finland and Taiwan [50,51]. Conversely, ischemic stroke can lead to AD [52,53], with previous studies establishing that microinfarcts are closely related to AD pathology [54,55]. Consequently, there is a correlation between AD and intracranial vessel arteriosclerosis [56,57]. Much of this correlation depends on platelet characteristics in AD and in older patients in general (reviewed in [2,58]). While we did not determine in this study’s experiments how Aβ is generated, we found blood clots inside the vessels, for which the platelets that are present could be the source of Aβ (Figure 1) and the reason why Aβ is present around the vessels (Figure 2). 

It has been reported that, while AD patients have similar platelet counts as age-matched controls, their platelets are in a more activated state [59]. There are clear age-related changes in platelet count, function, and reactivity, driven by changes in hematopoietic tissue, the composition of the blood, and vascular health, both in AD [2] and in stroke patients [60]. These age-related changes are particularly pertinent given that thrombotic diseases are much more prevalent in the elderly population [61]. As an example, acute coronary syndromes and atrial fibrillation—the most frequent indications for platelet inhibition or anticoagulation—occur mostly in older patients, as do stroke, transient ischemic attack, myocardial infarction, systemic embolism, deep-vein thrombosis, and pulmonary embolism [62]. Results from the literature and those reported here confirm that the tMCAO experimental procedure produces a similar overactivation of coagulation mechanisms, leading not only to blood clotting but also to massive generation of Aβ oligomers, thus resembling an early stage of AD pathology. Our results also fit the hypothesis that Aβ is a hitherto unrecognized natural antimicrobial agent of the innate immune system, which is related to the blood defense system that is normally activated during coagulation but also participates in a variety of pathological events related to platelet activation, including AD, glaucoma, brain cancer (see our review in [3]), and, according to this study, brain infarction.

## 5. Conclusions

As with focal ischemic stroke, after tMCAO many blood vessels in the damaged zone have coagulated blood containing partially degranulated platelets. In the damaged zone of the brain after tMCAO, Aβ is accumulated in blood vessel walls and nearby brain tissue. Tromethamine, a well-known reversible blocker of Aβ-formed pores, reduces the effects of tMCAO on the brain.

## Supplementary Materials

The following are available online at https://www.mdpi.com/2218-273X/9/8/350/s1: Figure S1, Control immunostaining for Aβ; Figure S2, Confocal 3D movie of Figure 2A panel 1; Figure S3, Confocal 3D movie of Figure 2A panel 2.
