# Supplementary figures and images for: Accumulation of Amyloid Beta (Aβ) Peptide on Blood Vessel Walls in the Damaged Brain after Transient Middle Cerebral Artery Occlusion
Source: Biomolecules. 2019 Aug 8;9(8):350. doi: 10.3390/biom9080350 (PMC6723874; doi:10.3390/biom9080350)

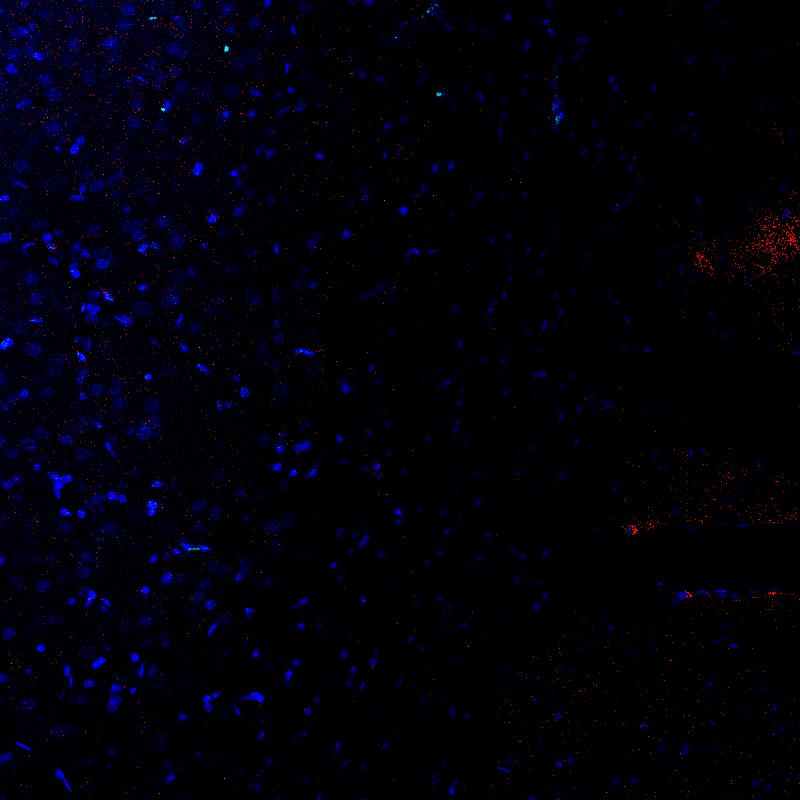

Supplement: Supplementary file 1 [file biomolecules-09-00350-s001.zip › S1.jpg]

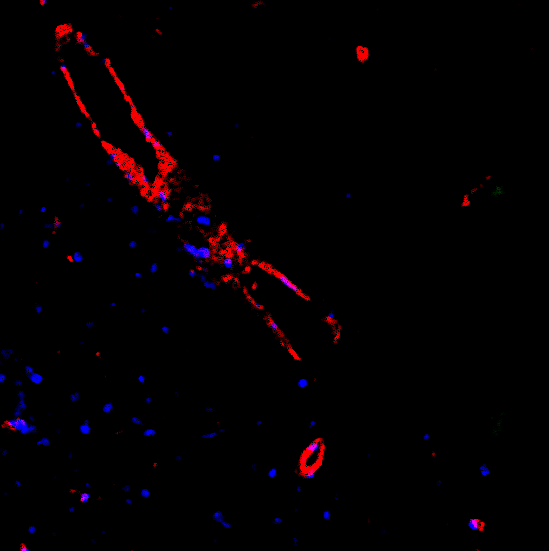

Supplement: Supplementary file 1 [file biomolecules-09-00350-s001.zip › S2.gif]

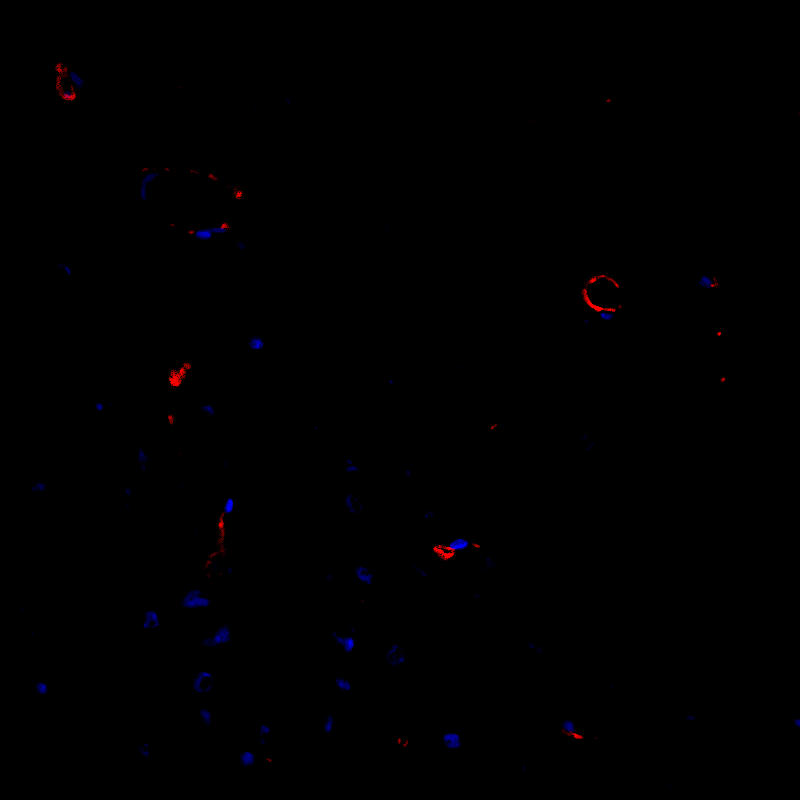

Supplement: Supplementary file 1 [file biomolecules-09-00350-s001.zip › S3.gif]
